# YAP1 facilitates the pathogenesis of psoriasis via modulating keratinocyte proliferation and inflammation

**DOI:** 10.1038/s41419-025-07521-3

**Published:** 2025-03-19

**Authors:** Cong Huang, Wenting Li, Changbing Shen, Bin Jiang, Kaoyuan Zhang, Xiahong Li, Weilong Zhong, Zizhuo Li, Zhenzhen Chen, Chaofeng Chen, Xingling Jian, Xiaoming Liu, Haiyan Huang, Lili Yang, Bo Yu

**Affiliations:** 1 Department of Dermatology, Skin Research Institute of Peking University Shenzhen Hospital, Peking University Shenzhen Hospital, Shenzhen Key Laboratory for Translational Medicine of Dermatology, Shenzhen Peking University - The Hong Kong University of Science and Technology Medical Center, Shenzhen, China; 2 Shenzhen Key Laboratory for Translational Medicine of Dermatology, Biomedical Research Institute, Shenzhen Peking University - The Hong Kong University of Science and Technology Medical Center, Shenzhen, China; 3 The Digestive and Reproductive System Cancers Precise Prevention Engineering Research Center of Jiangsu Province, Institute of Medicinal Biotechnology, Jiangsu College of Nursing, Huai’ an, Jiangsu China

**Keywords:** Mechanisms of disease, Chronic inflammation

## Abstract

Psoriasis is an autoinflammatory skin disease characterized by the abnormal activation of epidermal keratinocytes. The Hippo-YAP pathway is an evolutionarily conserved pathway that plays important roles in organ size control and tumorigenesis. Recently, accumulating evidence demonstrated that YAP1, the core downstream component of Hippo-YAP pathway, was up-regulated in psoriasis patients, suggesting its possible role in psoriasis development. However, its precise function and mechanism in psoriasis pathogenesis are still not well-clarified. In the present study, we confirmed the up-regulation of YAP1 in psoriasis keratinocytes by measuring its expression in psoriatic patient skins, psoriatic-like cellular model, and IMQ-induced mouse model. Further functional studies showed that YAP1 promoted keratinocyte proliferation and inflammation in vitro. Meanwhile, VP, a selective YAP1 antagonist, inhibited keratinocyte proliferation and inflammatory factor production in a dose-dependent way. Moreover, intradermal injection of si-Yap1 or VP hindered psoriasis development by impeding epidermal hyperplasia and relieving systemic inflammatory response in the IMQ-induced mouse model. Therefore, our findings suggest that YAP1 plays a crucial role in psoriasis pathogenesis through modulating keratinocyte activation and may serve as a novel target for the treatment of psoriasis.

## Introduction

Psoriasis is a recurring inflammatory skin disease. The global prevalence rate of psoriasis is around 3%, which seriously reduces the life quality of patients. The main pathological manifestations of psoriasis are abnormal proliferation and/or differentiation of keratinocytes, neovascularization in the epidermis, and infiltration of inflammatory cells into the skins [1, 2]. Although specific genetic and environmental factors play key roles in psoriasis pathogenesis, the precise molecular mechanism underlying is still unclear.

It is generally accepted that the dysregulated interplay between keratinocytes and the immune system represents one of the main causes for psoriatic epidermal hyperplasia [3–6]. The epidermal keratinocytes act as the “initiators” of psoriasis, upon stimulated, they proliferate rapidly and produce a variety of inflammatory factors, leading to the recruitment and activation of immune cells. The activated immune cells produce large numbers of pro-inflammatory mediators, including IL-17, TNF-α, and IL-22, which in turn result in the activation of keratinocytes and form a positive feedback loop, eventually causing the expansion of psoriasis plaques [7, 8]. Therefore, blocking the interaction between keratinocytes and immune cells in psoriasis patients holds potential for new drug development.

The Hippo-YAP pathway is a well-known signaling pathway in controlling organ size and tumorigenesis [9–11]. Once activated, the upstream kinases of the pathway, including MST1/2 and Sav1, form a kinase complex and lead to the phosphorylation of LATS1/2. Phosphorylated LATS1/2 further promotes the phosphorylation of the main downstream effectors (YAP1 and TAZ), leading to their cytoplasmic retention or ubiquitination-dependent degradation [10, 12]. The non-phosphorylated YAP/TAZ will be translocated to the nucleus and bind to specific transcription factors, thereby driving the expression of genes related to proliferation, differentiation, and inflammation [13, 14].

In addition to the tumorigenic role [15–17], recent studies have shown that the Hippo pathway is dysregulated in psoriatic skin and may be involved in psoriasis pathogenesis. For instance, Tang et al. reported that MST1 (the upstream kinase of the Hippo pathway) was significantly up-regulated in the skin lesions and T cells of psoriasis patients. Meanwhile, MST1 promotes the proliferation and activation of T cells, suggesting its role in psoriasis pathogenesis [18]. In addition, Ren et al. found that TEAD4, an important downstream transcription factor of the Hippo pathway, was dramatically up-regulated in the psoriatic lesions. Further studies showed that TEAD4 regulates the interaction between keratinocytes and T cells by modulating the cytokine secretion in psoriatic keratinocytes [19]. Despite the widely dysregulated Hippo components in psoriasis patients, the specific mechanism that Hippo-YAP regulates the psoriasis pathogenesis still needs to be further explored.

YAP1, the core downstream modulator of the Hippo-YAP pathway, has been shown to play crucial roles in keratinocyte proliferation. For example, Yap1 knockdown inhibits, whereas Yap1 activation promotes the stem/progenitor cell expansion in the epidermis of mouse skin [20]. Additionally, blocking YAP1 in human keratinocytes and mouse skins by TEADi results in reduced keratinocyte proliferation [21]. Interestingly, YAP regulates the pro-inflammatory response in endometrial cancer cells through NF-κB and IL-6 signals [22], both of which are key regulators of psoriasis inflammation [23–25], suggesting the potential role of YAP1 in psoriatic pathogenesis.

By immunohistochemical staining, Jia et al. revealed that YAP1 was up-regulated in psoriatic lesions [26]. Our preliminary data further confirmed that psoriatic inflammation results in the up-regulation of YAP1: (1) At the cellular level, we used M5 (TNF-α, IL-17, IL-22, IL-1α, and OSM, abbreviated as M5) to induce a psoriatic-like inflammatory state in keratinocytes (KC), and found that M5 induction significantly increased YAP1 expression in KCs; (2) In the imiquimod (IMQ)-induced mouse model, we found that Yap1 was dramatically elevated in the IMQ-treated mouse epidermis, suggesting that the inflammatory factors up-regulate YAP1 expression and the abnormal expressed YAP1 may associate with psoriasis pathogenesis.

Our mechanistic study showed that YAP1 contributes to psoriasis pathogenesis by promoting the proliferation and inflammation of keratinocytes. Importantly, knockdown/pharmacological blocking of Yap1 inhibited the immunopathological changes and prevented the psoriasis development in the IMQ-induced mouse model. Therefore, YAP1 represents a novel therapeutic target for the treatment of psoriasis.

## Material and methods

### Patients and specimen collection

All the patients and healthy donors included in this study (Six psoriasis patients and five healthy donors) were recruited from the Department of Dermatology, Peking University Shenzhen Hospital (Shenzhen, China). The psoriasis patients enrolled in this study were not treated with topical therapy for at least two weeks or received systemic treatment for at least one month before the skin biopsy. The PASI score was recorded for each psoriasis patient before the biopsy. Biopsies (6 mm) were taken from psoriasis patients at the lesional site. Biopsies collected from the non-inflamed skin of healthy donors were used as controls. The specimens were divided into two parts, one part was immediately placed in liquid nitrogen for RNA extraction, while the other part was fixed with 4% paraformaldehyde for tissue sectioning. The present study was approved by the Ethics Committee of Peking University Shenzhen Hospital, which was conducted in accordance with the Declaration of Helsinki. Informed consents were obtained from all participants.

### Cell lines, antibodies, siRNAs, chemicals, and reagents

HaCaT cells were from China Center for Type Culture Collection (Wuhan, China) and kept in our own lab. HaCaT cells were cultured in DMEM (Gibco, Carlsbad, CA, USA) supplemented with 10% fetal bovine serum (Atlanta Biologicals, Lawrenceville, GA), 1 × penicillin, and 1 × streptomycin. Cells were incubated at 37°C in a humidified condition supplemented with 5% CO_2_. Antibodies against GAPDH (AF1186), phospho-NF-κB p65 (Ser311) (AF5878), NF-κB p65 (AF1234), and IL-1β (AF7209) were from Beyotime Biotechnology (China), CTGF (25474-1-AP), ANKRD1 (11427-1-AP), and CYR61 (26689-1-AP) antibodies were from Proteintech (Rosemont, IL), while phospho-STAT3 (Tyr705) (#9145), phospho-STAT3 (Ser727) (#9134), STAT3 (#30835), and YAP1 (#4912) antibodies were from Cell Signaling Technology Inc. (Danvers, MA). Secondary antibodies for Western blot and immunohistochemistry/immunofluorescence analysis were from Beyotime Biotechnology (China). si-YAP1 and indicated si-CTL were from RiboBio Inc. (Ribobio, Guangzhou, China). RT-qPCR-related chemicals or reagents were from Bio-Rad (Hercules, CA). The SuperSignal West Femto Chemiluminescent Substrate Kit was from Thermo Scientific (Rockford, IL). Verteporfin (VP) was from Selleck Chemicals (#S1786). All other molecular- grade chemicals were purchased from Sigma (St. Louis, MO) or Fisher (Pittsburgh, PA) unless otherwise mentioned.

### Western blotting

Western blot was performed as described previously [27, 28]. Briefly, samples were harvested using ice-cooled RIPA lysis buffer pre-mixed with protease and phosphatase inhibitor cocktails. Samples (30 µg protein) were loaded to a 12% SDS-PAGE gel, fractioned through electrophoresis, and transferred onto PVDF membranes. The membranes were blocked with 5% BSA, and then incubated with appropriate primary and secondary antibodies. The immunosignal was detected using the SuperSignal West Femto Chemiluminescent Substrate Kit. The images were captured and analyzed using Bio-rad imaging system (Hercules, CA).

### Immunohistochemistry/Immunofluorescence staining

YAP1 expression was detected by immunohistochemistry staining, while STAT3, NF-κB, and Ki67 were detected using immunofluorescence staining as described previously [27, 28]. Briefly, tissues were deparaffinized with xylene, rehydrated with graded ethanol series, and then autoclaved in an unmasking solution (Vector Laboratories, Burlingame, CA) for antigen retrieval. For IHC staining, tissues were blocked with 3% hydrogen peroxide at room temperature for 20 min. All the tissues were then blocked with blocking buffer at room temperature for 1 h followed by incubation with primary antibodies at 4 °C overnight. Biotinylated/Fluorescence-labeled secondary antibodies (Beyotime, China) were added and incubated for 30 min at room temperature. For IHC staining, the immunosignal was visualized using a DAB kit from Cell Signaling Technology Inc. (#8059, Danvers, MA). The sections were counterstained with Mayer’s hematoxylin for IHC staining, while counterstained with DAPI for IF staining. The images were scanned and captured using a PANNORAMIC™ Digital Slide Scanner (3DHISTECH Ltd., Hungary).

### Reverse transcription-quantitative PCR

Reverse transcription-quantitative PCR (RT-qPCR) was used to determine the mRNA expression. Total RNA was prepared with TRIzol reagent (Invitrogen; Carlsbad, CA). RNA concentration was determined using Nanodrop-2000 (Thermo Scientific, Carlsbad, CA). Reverse transcription was done by using a high-capacity cDNA reverse transcription kit (Ribobio, Guangzhou, China). The RT-qPCR amplification was performed in a Bio-Rad CFX96 real-time fast PCR system (Bio-Rad, Hercules, CA), and the relative expression of the objective gene was calculated using the 2^−ΔΔCt^ method. All the primers used in this study were designed using the online software PrimerBank (https://pga.mgh.harvard.edu/primerbank/index.html).

### Cell transfection, VP treatment, M5 treatment, cell proliferation, and cell cycle assay

For the gene knockdown assay, HaCaT cells were cultured to 70% confluent and then transfected with YAP1 siRNA and indicated siRNA control using RNAiMAX for 24 h according to the manufacturer’s instruction. For the overexpression assay, we used Lipofectamine 3000 Transfection Reagent to deliver plasmid or vector into cells for 24 h. For the verteporfin (VP) treatment assay, cells were incubated with VP (HY-B0146, MedChemExpress) of different working concentrations (5 and 10 µM), or DMSO of equal volume for 24 h. M5 treatment was conducted, followed by cell transfection and/or VP treatment. For the M5 treatment assay, cells were incubated within M5 cocktail, containing recombinant human IL-1α (#200-01 A), IL-17A (#200-17), IL-22 (#200-22), oncostatin M (#300-10), and TNF-α (#300-01 A) (all of the five inflammatory factors were brought from Peprotech and the working concentration for each inflammatory factor was 10 ng/mL), or PBS of equal volume for 24 h. Cell proliferation assay was conducted as previously described [27]. Cell cycle assay was used to detect the cell phase distribution in different groups [29]. Briefly, trypsinized cells were fixed in pre-cooled 70% ethanol at 4 °C for 30 min. Following incubation in 50 mg/mL propidium iodide solution for 30 min at room temperature, the cells were tested with a flow cytometry instrument (FACS Calibur; Becton Dickinson, USA), and data were analyzed using the MODFIT software (Verity Software House, USA).

### IMQ-induced psoriasis-like mouse model and In vivo si-Yap1/Verteporfin (VP) treatment

Female C57BL/6 mice (7 weeks of age) were kept under controlled conditions. To construct the IMQ-induced psoriasis-like mouse model, the mice were treated with a daily topical dose of 62.5 mg IMQ cream (5%, Sichuan Med-shine Pharmaceutical, H20030128) on the shaved back for 5 consecutive days. Control mice were treated with the same dose of vehicle cream. For the in vivo si-Yap1/Verteporfin (VP) treatment assay, female C57BL/6 mice (7 weeks of age) were intradermally injected with si-Yap1 or Verteporfin, as well as their corresponding controls, on day -2 and day -1 before the application of IMQ. The mice were then treated with IMQ cream daily for 5 consecutive days. Si-Yap1, as well as its control, were synthesized by Guangzhou RiboBio Co., Ltd. The oligonucleotide sequences that target Yap1 are shown as follows: 5’-CCACCAAGCTAGATAAAGA-3’. During the process of si-Yap1/VP treatment, the PASI (Psoriasis Area and Severity Index) scores of the mice were calculated daily as previously described [27]. For si-Yap1/VP treatment, all mice were sacrificed on the fifth day, and tissues of the mouse skins were collected (one part was quickly placed in liquid nitrogen for RNA/protein extraction, and the other part was fixed with 4% paraformaldehyde for tissue sectioning). All procedures were approved and supervised by Shenzhen Perking University - Hong Kong University of Science and Technology Medical Center Animal Care and Use Committee.

### Isolation of mouse epidermis

Mouse epidermal tissues were isolated as previously described [30]. Initially, the subcutaneous adipose tissues and dermis layer were removed from the skin specimens of mice. The remaining skin tissues were then cut into 1 cm × 1 cm sections and incubated in Dispase II at 4 °C overnight. To collect epidermal samples for RNA and/or protein analysis, the dermis was separated from the epidermis, then the epidermis was gently washed in PBS for three times, and stored in liquid nitrogen.

### Microarray data processing and validation of *YAP1* expression

A gene expression profile of psoriasis, namely GSE13355, was obtained from the GEO database [31] (https://www.ncbi.nlm.nih.gov/geo/). 64 punch biopsies of normal healthy skin (Normal skin from controls, NN), 58 psoriatic lesions (Involved skin lesions from psoriatic patients, PP), and 58 samples from individual-matched uninvolved skin of psoriatic patients (Uninvolved skin from psoriatic patients, PN) were used for further analysis. After collecting the rare data from GSE13355, we validated the mRNA expression of *YAP1* in these samples using the R software.

### Statistics

All experiments were repeated at least three times unless otherwise noted. Data are presented as mean ± SEM. Statistical analysis was conducted using GraphPad Prism software (GraphPad Software, Inc. La Jolla, CA). Data were analyzed for significance using one-way ANOVA with Tukey’s post-hoc tests. A value of *P* < 0.05 was considered statistically significant.

## Results

### YAP1 is up-regulated in psoriatic keratinocytes

**Fig. 1:**
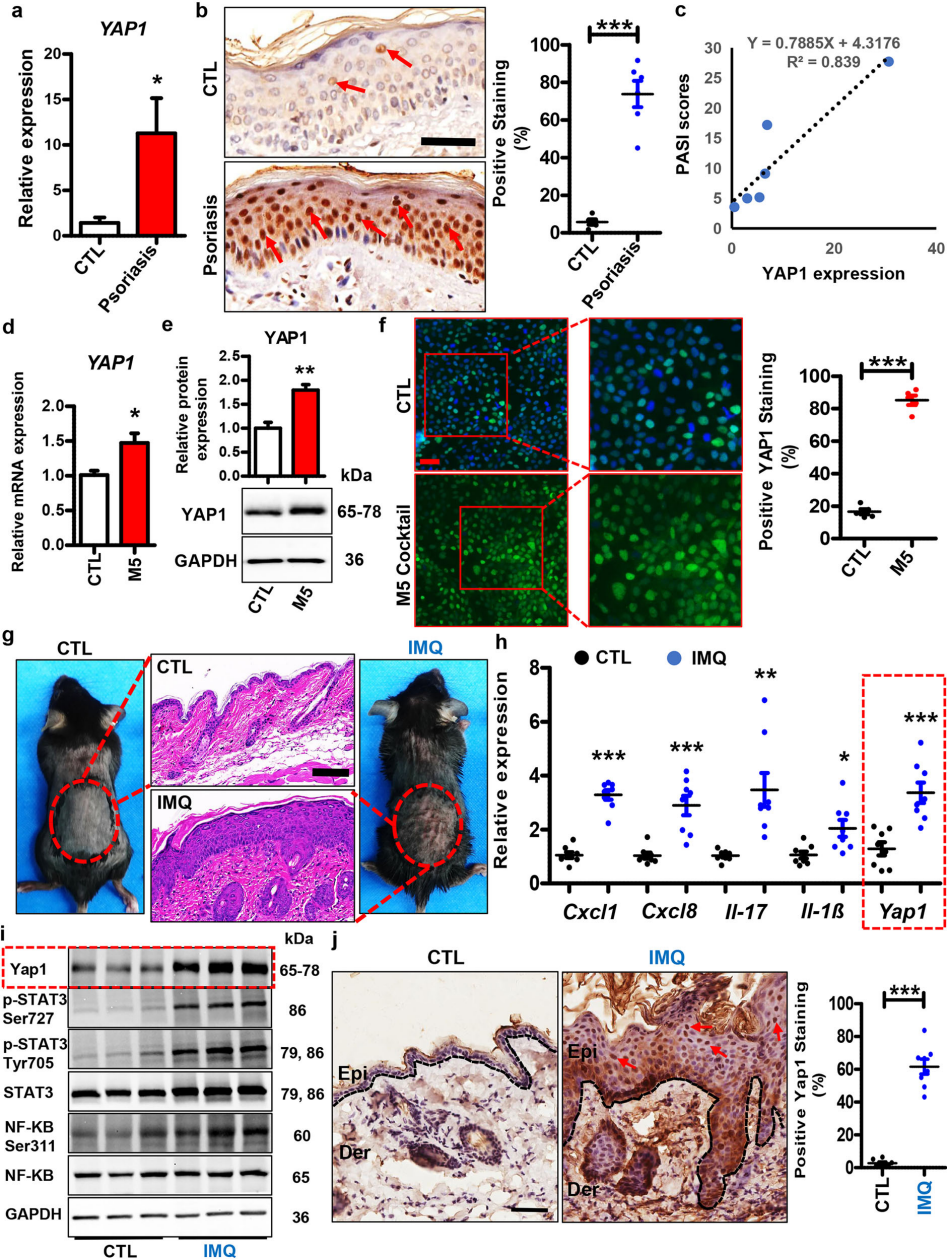
YAP1 is up-regulated in psoriasis patients, M5-induced keratinocytes, and IMQ-induced mouse skins. **a** mRNA expression of *YAP1* in skins derived from healthy donors (CTL, *n* = 5) and psoriasis patients (Psoriasis, *n* = 6). **b** Left panel: Representative immunohistochemistry images showing YAP1 expression in skins derived from psoriasis patients and healthy donors. Red arrows indicate the positive staining cells. Scar bar: 200 µm. Right panel: Quantification of YAP1 positivity in clinical samples. **c** Correlation of YAP1 expression in psoriatic patients with patient PASI scores. **d**, **e** mRNA and protein expression of YAP1 in HaCaT cells with or without M5-treatment (*n* = 3). **f** Representative immunofluorescence images showing the expression of YAP1 in HaCaT cells treated with M5/Control. Scar bar: 50 µm. Quantification of YAP1 positive cells in HaCaT cells with different treatments (*n* = 5). **g** Representative images showing the skin lesions and histology features of CTL (Vaseline)- and IMQ-treated mice. Scar bar: 100 µm. **h** mRNA levels of *Yap1* and psoriasis-related genes in the epidermis of CTL- and IMQ- mice (*n* = 8). **i** Representative blots showing protein levels of Yap1 and inflammatory-related pathways in the epidermal tissues of CTL- and IMQ- mice. **j** Left panel: Yap1 levels were analyzed in the CTL- and IMQ- mice using immunohistochemistry. Scar bar: 50 µm. Right panel: Quantification of Yap1 positivity in the mouse epidermis of different groups (*n* = 8). ***P < 0.001, **P < 0.01, *P < 0.05, compared with the indicated controls.

It has been reported that YAP1 is up-regulated in psoriatic lesions compared to healthy skins [26, 32]. In the present study, we first validated the up-regulation of YAP1 in psoriasis lesions using psoriatic and healthy skin collected from our department. As shown in Fig. 1a, *YAP1* mRNA was dramatically increased in the psoriasis lesions collected by our dermatologists. Meanwhile, the IHC analysis showed that YAP1 proteins were significantly up-regulated in psoriasis lesions than healthy controls (Fig. 1b). More importantly, a positive correlation between YAP1 expression and patient PASI scores was observed in these clinical samples (Fig. 1c). To further explore the possible role of YAP1 in psoriasis pathogenesis, we examined its expression in psoriasis lesional and non-lesional skins. By analyzing the GEO database, we found that *YAP1* was significantly elevated in the psoriasis lesional skins, compared to the non-lesional skins (Fig. S1). Thus, YAP1 is elevated in psoriatic lesions and may affect the disease severity.

To figure out whether YAP1 is dysregulated by psoriasis-related inflammatory factors in vitro, we treated HaCaT cells with M5 to induce psoriatic-like inflammation in cultured keratinocytes. Interestingly, both the mRNA (Fig. 1d) and protein levels (Fig. 1e) of YAP1 were increased in M5-treated keratinocytes compared to the control group. Additionally, increased YAP1 stainings were observed in HaCaT cells treated with M5 inflammatory factors (Fig. 1f), meaning that psoriatic inflammation leads to YAP1 up-regulation in keratinocytes.

To further evaluate the expression profile of Yap1 in vivo, mice were topically treated with IMQ/Vaseline, as mentioned in the Material and Method section. As expected, IMQ-treated mice showed increased skin inflammation, while Vaseline-treated mice (CTL group) did not show apparent signs of skin lesions (Fig. 1g). We then isolated the epidermal tissues from IMQ/CTL-induced mice, and performed RT-qPCR analysis and western blotting assay to detect the mRNA and protein levels of Yap1 in the epidermis. As demonstrated in Fig. 1h, the mRNA expression of psoriasis-related genes, as well as *Yap1* gene, was obviously elevated in the IMQ-treated mouse epidermis (Fig. 1h, marked in a red box). Moreover, as shown in Fig. 1i, the blotting results demonstrated that the inflammatory-related pathways were significantly activated in the IMQ-induced epidermis, as compared to the CTL epidermis (Fig. 1i). Additionally, Yap1 proteins were dramatically up-regulated in the IMQ-induced epidermis than the CTL epidermis (Fig. 1i, marked in a red box). Further, immunohistochemistry results suggested that the psoriatic inflammatory environment promoted Yap1 protein expression in the IMQ-induced epidermis (Fig. 1j).

All the aforementioned results strongly suggest that YAP1 is up-regulated in psoriasis keratinocytes and it plays a role in psoriatic pathogenesis.

### The regulatory role of YAP1 in keratinocyte proliferation

**Fig. 2:**
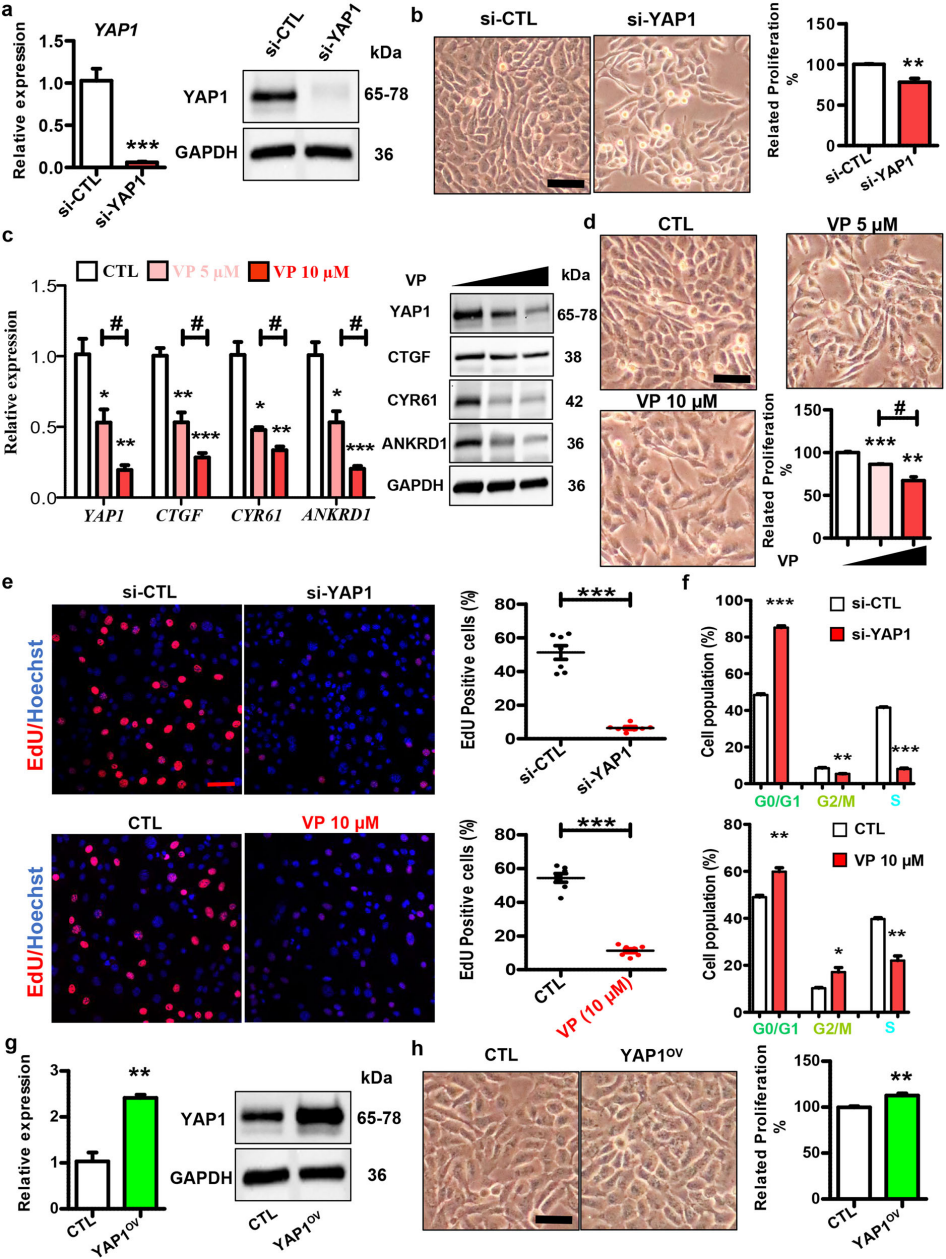
Function of YAP1 in keratinocyte proliferation. **a** Expression of YAP1 in HaCaT cells transfected with si-YAP1/si-CTL. mRNA levels were analyzed using RT-qPCR (*n* = 4), while protein levels were detected by western blotting. **b** Left panel: Representative images showing cell number changes of HaCaT cells transfected with si-YAP1/si-CTL. Scar bar: 50 µm. Right panel: Relative proliferation rate of HaCaT cells transfected with si-YAP1/si-CTL (*n* = 4). **c** Left panel: mRNA expression of *YAP1* and its downstream target genes in HaCaT cells treated with VP of different concentrations or CTL (*n* = 3). Right panel: Representative blotting showing protein levels of YAP1 and its downstream targets in HaCaT cells treated with VP of different concentrations. **d** Representative images showing the cell number changes of HaCaT cells treated with VP of different concentrations or CTL. Scar bar: 50 µm. Graph showing relative proliferation rate of HaCaT cells treated with VP/CTL (*n* = 3). **e** Cellular proliferation of keratinocytes from different groups (si-YAP1/si-CTL and VP/CTL) was measured by EdU assay (*n* = 7). Scar bar: 50 µm. **f** Cell cycle was tested using flow cytometry after different treatments (si-YAP1/si-CTL and VP/CTL). The ratios of cells in different phases were calculated (*n* = 3). **g** mRNA and protein expression of YAP1 in HaCaT cells transfected with YAP1 overexpression/Control vector (*n* = 3). **h** Representative images showing cell number changes of HaCaT cells transfected with YAP1 overexpression/CTL vector (Left panel). Scar bar: 50 µm. The relative proliferation rate of HaCaT cells transfected with YAP1 overexpression/CTL vector (*n* = 3) (Right panel). #*P* < 0.05, ***P < 0. 001, **P < 0.01, *P < 0.05, compared with the indicated controls.

To explore the possible role of YAP1 in vitro, we used synthetic si-YAP1 to knockdown YAP1 in HaCaT cells (Fig. 2a). Interestingly, YAP1 knockdown led to decreased keratinocyte proliferation (Fig. 2b), suggesting its contributory role to epidermal hyperplasia, which is one of the main characteristics for psoriasis. We then used verteporfin (VP), a selective YAP1 antagonist [33], to pharmacologically inhibit its activity in HaCaT cells. As shown in Fig. 2c, VP treatment dramatically decreased the expression of YAP1 and its downstream target genes in a dose-dependent manner. Additionally, VP treatment significantly inhibited keratinocyte proliferation in a dose-dependent way (Fig. 2d). Moreover, the EdU staining results demonstrated that the inhibition of YAP1 blocked the proliferation of HaCaT keratinocytes (Fig. 2e). Mechanistically, both si-YAP1 and VP treatment arrested keratinocytes in the G0/G1 phase (Fig. 2f). Thus, YAP1 may promote keratinocyte proliferation through regulating cellular cycle.

To further confirm the role of YAP1 in keratinocyte proliferation, we transfected HaCaT cells with YAP1 overexpression vector to force the exogenous YAP1 expression in keratinocytes (Fig. 2g). As shown in Fig. 2h, YAP1 overexpression significantly promoted keratinocyte proliferation. Therefore, YAP1 contributes to keratinocyte hyperproliferation.

### The regulatory role of YAP1 in keratinocyte inflammation

**Fig. 3:**
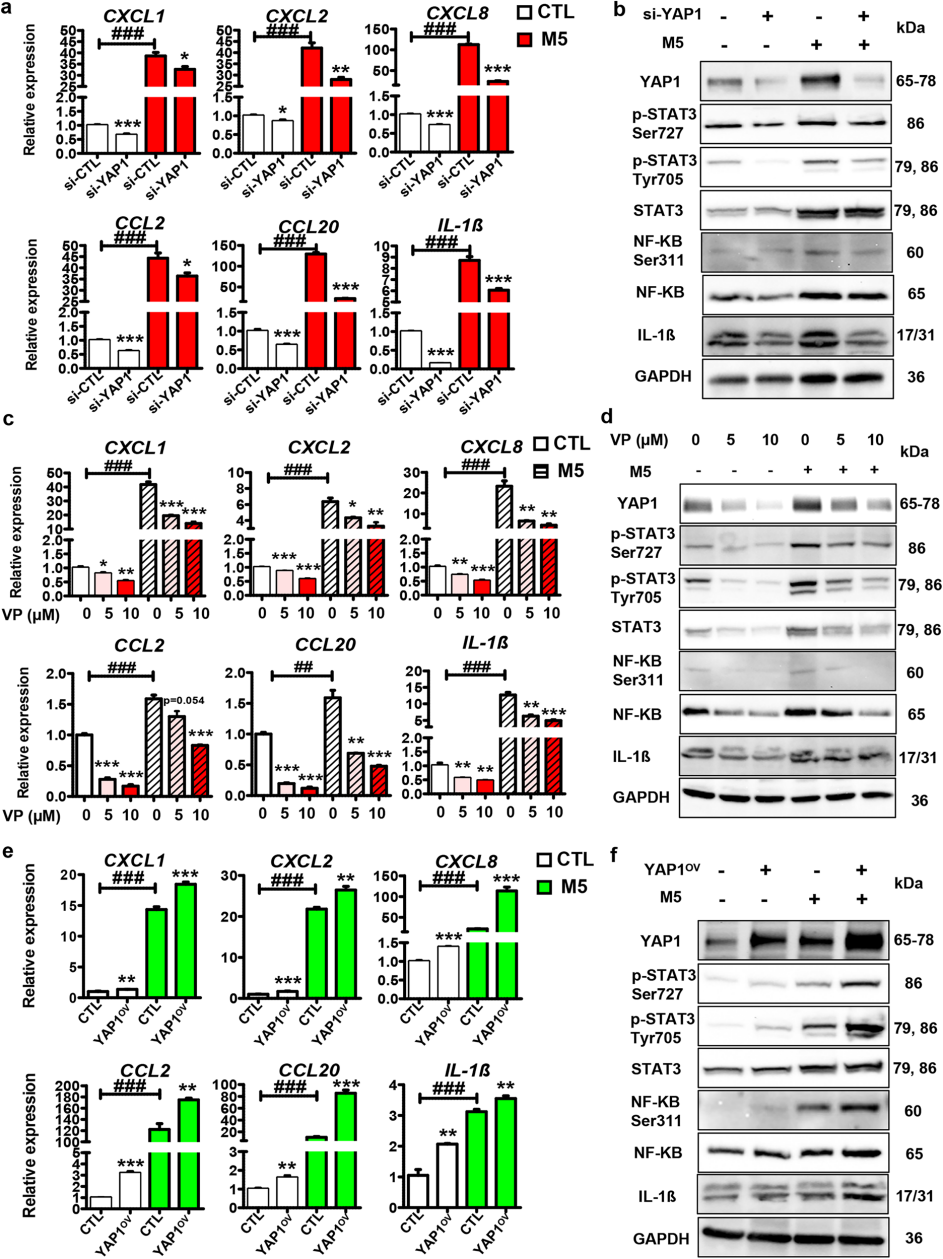
Function of YAP1 in keratinocyte inflammation. **a** mRNA levels of inflammatory-related genes, including *CXCL1*, *CXCL2*, *CXCL8*, *CCL2*, *CCL20*, and *IL-1β* in HaCaT cells transfected with si-YAP1/si-CTL in the presence or absence of M5 treatment (*n* = 4). **b** Representative blots showing protein levels of YAP1 and inflammatory-related signals, including phospho-STAT3 (Ser727), phospho-STAT3 (Tyr705), phospho-NF-κB p65 (Ser311), and IL-1β et al. in HaCaT cells transfected with si-YAP1/si-CTL in the presence or absence of M5 treatment. **c**, **d** mRNA levels of inflammatory-related genes and protein levels of inflammatory-related signals in HaCaT cells treated with VP of different concentrations in the presence or absence of M5 treatment (*n* = 4). **e**, **f** mRNA levels of inflammatory-related genes and protein levels of inflammatory-related signals in HaCaT cells transfected with YAP1^OV^ or CTL in the presence or absence of M5 treatment (*n* = 4). ###*P* < 0.001, ##*P* < 0.01, ***P < 0.001, **P < 0.01, *P < 0.05, compared with the indicated controls.

Keratinocyte inflammation plays an important role in psoriasis development [3–8]. To explore the possible role of YAP1 in keratinocyte inflammation, we used si-YAP1 or VP to knockdown/block YAP1 in HaCaT cells. Meanwhile, M5 was introduced into our system to induce the psoriatic inflammatory phenotype of HaCaT cells. Unexpectedly, si-YAP1 led to the down-regulation of inflammatory genes, including *CXCL8*, *CCL20*, and *IL-1β*, in the presence or absence of M5 treatment (Fig. 3a). Interestingly, the protein levels of phospho-STAT3 (Ser727), phospho-STAT3 (Tyr705), phospho-NF-κB p65 (Ser311), and IL-1β were dramatically decreased by si-YAP1 (Fig. 3b), meaning that YAP1 knockdown results in the disruption of the STAT3 and NF-κB inflammatory pathways. Pharmacological inhibition assay confirmed that VP (YAP1 inhibitor) treatment decreased the expression of inflammatory genes (Fig. 3c), as well as the inflammatory pathways (Fig. 3d) in a dose-dependent manner in keratinocytes.

To further confirm the regulatory role of YAP1 in keratinocyte inflammation, we performed the gain-of-function assay in HaCaT cells using YAP1 overexpression vector in the presence or absence of M5 treatment. As shown in Fig. 3e, YAP1 overexpression significantly promoted the expression of inflammatory-related genes in the presence or absence of M5 treatment. Moreover, the STAT3 and NF-κB pathways were obviously activated by YAP1 overexpression (Fig. 3f). All these results indicate that YAP1 promotes the inflammatory response of keratinocytes.

### The role of Yap1 in the IMQ-induced mouse model

**Fig. 4:**
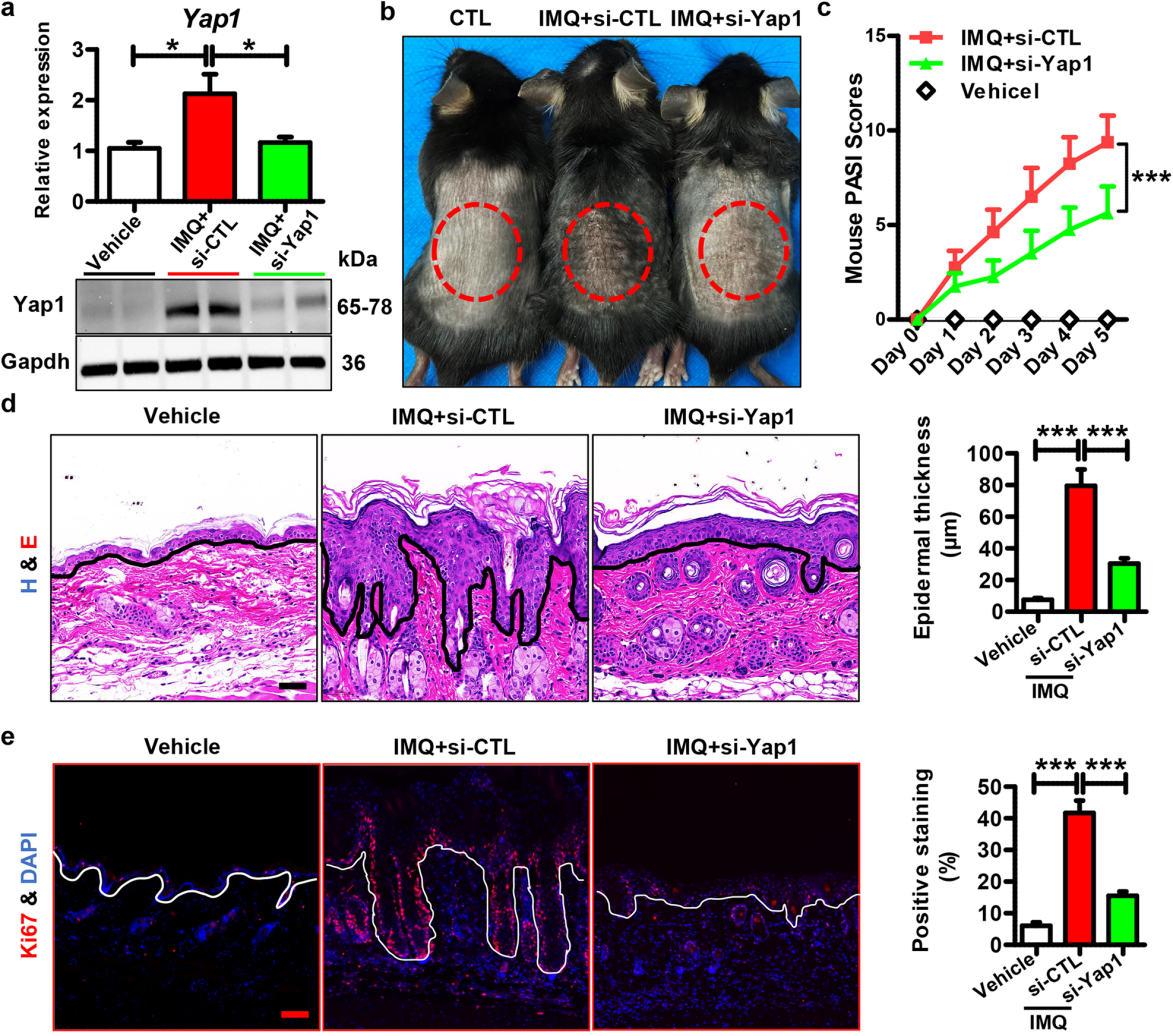
Function of si-Yap1 in epidermal hyperplasia in IMQ-induced mouse model. **a** Expression levels of Yap1 in the mouse epidermis of different groups. Vehicle means mice injected with si-CTL and treated with Vaseline; IMQ + si-CTL and IMQ + si-Yap1 mean mice injected with si-CTL and si-Yap1, respectively, then topically treated with IMQ. mRNA levels were analyzed using RT-qPCR (*n* = 8), while protein levels were detected by western blotting. **b** Representative pictures of the mouse skins from different groups. **c** PASI scores of mouse skin lesions in different groups at different time points (*n* = 8). **d** Histology features of skin tissues derived from different groups and quantification data of epidermal thickness in the mouse skins of each group (*n* = 8). Scar bar: 25 µm. **e** Representative images showing the Ki67 expression in mouse skins of different groups and quantification data of Ki67 positive cells in the mouse skin samples of each group (*n* = 8). Scar bar: 25 µm. ***P < 0.001, *P < 0.05, compared with the indicated controls.

To confirm the effects of YAP1 in psoriasis development, we intradermally injected synthetic si-Yap1 into the shaved back of mice to knockdown Yap1 in vivo. Then, mice were topically treated with IMQ/Vaseline. As expected, Yap1 was significantly up-regulated in the IMQ + si-CTL group, compared to the vehicle group (Vaseline + si-CTL), whereas si-Yap1 administration dramatically decreased its expression (Fig. 4a & S2). Meanwhile, the IMQ-treated mice showed increased skin lesions and higher PASI scores than the vehicle group, whereas a significant decrease in skin inflammation and severity score was observed following si-Yap1 treatment (Fig. 4b, c). Additionally, histological analysis (Fig. 4d) and Ki67 staining (Fig. 4e) showed that IMQ induced thickened and hyperplastic epidermis, whereas si-Yap1 decreased the IMQ-induced excessive proliferation of epidermal keratinocytes. These results demonstrate that Yap1 knockdown blocks the epidermal hyperplasia in vivo.

**Fig. 5:**
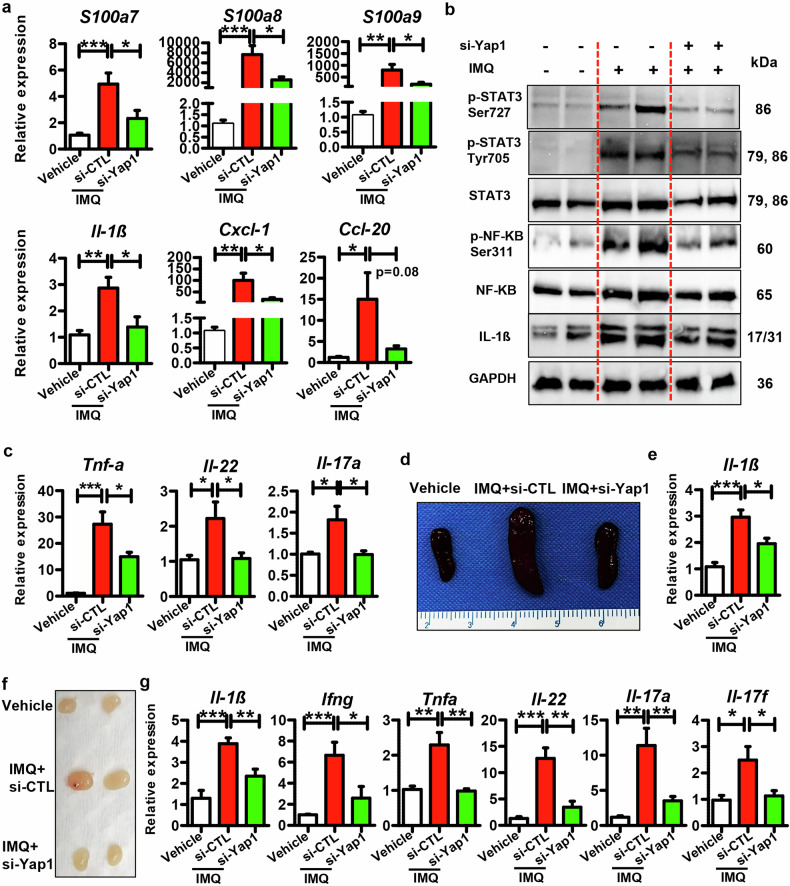
Function of si-Yap1 in regulating cutaneous inflammation and systemic inflammatory response in IMQ-induced mouse model. **a** mRNA levels of psoriasis-related genes and inflammatory genes in the skin tissues derived from different groups. **b** Representative blots showing protein levels of inflammatory-related signals, including phospho-STAT3 (Ser727), phospho-STAT3 (Tyr705), phospho-NF-κB p65 (Ser311), and Il-1β, et al. in the skin tissues derived from different groups. **c** mRNA levels of *Tnfa*, *Il-22*, and *Il-17* in the mouse skins of different groups. **d** Representative pictures showing the spleen sizes of mice from different groups. **e** mRNA levels of *Il-1β* in spleens derived from mice of different groups. **f** Representative images showing the lymph node sizes of mice from different groups. **g** mRNA levels of *Il-1β*, *Ifng*, *Tnfa*, et al. in lymph nodes derived from mice of different groups. *n* = 8 for each group. ***P < 0.001, **P < 0.01, *P < 0.05 compared with the indicated controls.

In addition, si-Yap1 decreased psoriasis-related genes in the mouse skins topically administrated with IMQ (Fig. 5a). Moreover, si-Yap1 suppressed the expression of inflammatory genes as well (Fig. 5a). Accordingly, si-Yap1 inhibited the STAT3 and NF-κB inflammatory pathways in the mouse skins treated with IMQ (Fig. 5b), which was further confirmed by the IF staining results (Fig. S3). These results indicate that Yap1 knockdown decreases the epidermal inflammation in vivo.

More importantly, si-Yap1 significantly decreased *Tnfα*, *Il17a*, and *Il22* expression in mouse skins (Fig. 5c), suggesting a hindered inflammatory response. Notably, the spleen size and splenic *Il-1β* expression in the si-Yap1 administration group dramatically decreased, compared to the IMQ + si-CTL group (Fig. 5d, e), indicating that si-Yap1 helps to relieve the systemic inflammatory response in the IMQ-treated mice. Meanwhile, decreased lymph node size was observed in the IMQ + si-Yap1 group (Fig. 5f). Moreover, the mRNA levels of *Ifng*, *Tnfα*, *Il17*, and *Il22* in the lymph nodes were suppressed by si-Yap1 administration (Fig. 5g), confirming that si-Yap1 systemically reduces the inflammatory response in the IMQ-induced mice. Thus, Yap1 knockdown inhibits the immunopathological changes and blocks psoriasis development in vivo.

### The therapeutic effects of VP on IMQ-induced mouse model

**Fig. 6:**
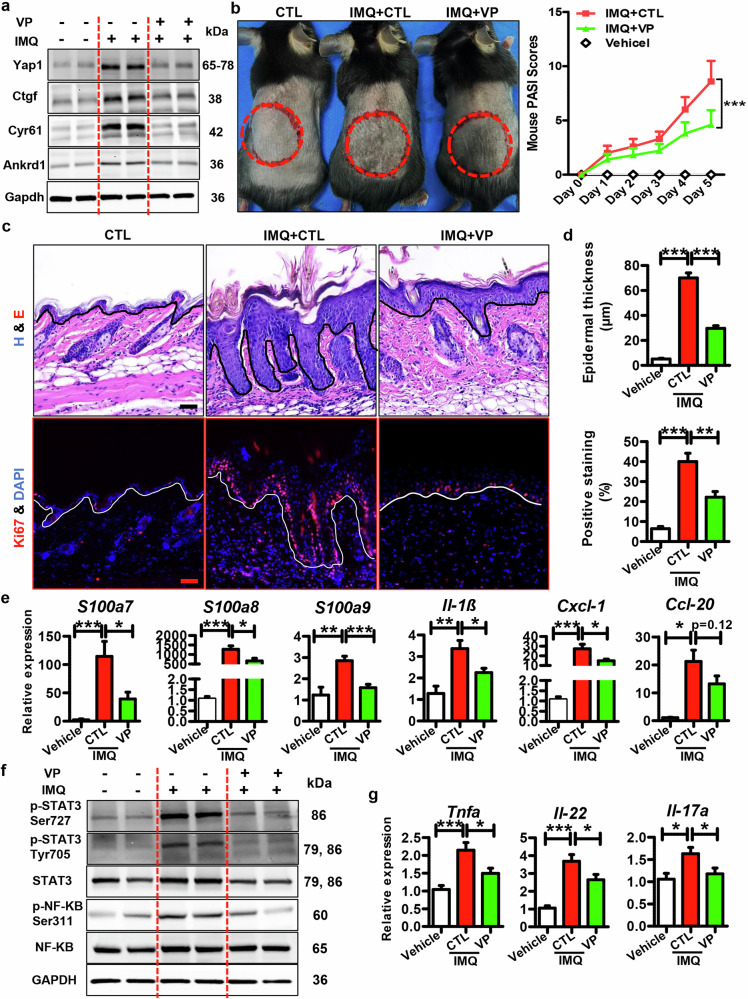
The therapeutic effects of VP on IMQ-induced mouse model. **a** Representative blots showing the protein expression of Yap1 and its downstream targets in mouse epidermis from different groups. **b** Left panel: Representative pictures showing the mouse skins treated with VP/CTL in the presence or absence of IMQ treatment. Right panel: PASI scores of mouse skins in different groups at different time points. **c** Histology features (upper panel) and Ki67 staining (lower panel) of mouse skins derived from different groups. Scar bar: 25 µm. **d** Quantification data of skin epidermal thickness (upper panel) and Ki67 positive cells (lower panel) in the mouse skins of different groups. **e** mRNA levels of psoriasis-related genes and inflammatory genes in the skins derived from different groups (*n* = 8). **f** Representative blots showing protein levels of inflammatory-related signals, including phospho-STAT3 (Ser727), phospho-STAT3 (Tyr705), and phospho-NF-κB p65 (Ser311) et al. in the skin tissues derived from different groups. **g** mRNA levels of Th cell markers in the skins derived from different groups (*n* = 8). ***P < 0.001, **P < 0.01, *P < 0.05 compared with the indicated controls.

We further assessed the therapeutic effects of VP on the psoriasis mouse model. VP was intradermally injected into mouse skins, and mice were then topically treated with IMQ/Vaseline. As shown in Fig. 6a, VP treatment significantly decreased the expression of Yap1 and its downstream targets in the epidermal tissues of IMQ-induced mice. Meanwhile, VP treatment dramatically decreased the skin inflammation and severity score in the presence of IMQ treatment (Fig. 6b). Additionally, the histological analysis and Ki67 staining showed that VP suppressed the IMQ-induced excessive proliferation of keratinocytes (Fig. 6c, d). Moreover, VP decreased the expression of psoriasis-related genes and inflammatory genes in the presence of IMQ treatment (Fig. 6e). Accordingly, VP treatment inhibited the STAT3 and NF-κB inflammatory pathways in the mouse skins treated with IMQ (Fig. 6f). Furthermore, VP suppressed the expression of *Tnfα*, *Il17a*, and *Il22* in mouse skins (Fig. 6g). These results suggest that VP treatment alleviates the skin inflammation in vivo. Importantly, VP suppressed the systemic inflammatory response in IMQ-treated mice, as indicated by a decreased spleen size (Fig. S4a), suppressed splenic *Il-1β* expression (Fig. S4b), and decreased *Ifng*, *Tnfα*, *Il17*, as well as *Il22* expression in the lymph nodes following VP administration (Fig. S4c).

In summary, VP impedes the psoriasis development in IMQ-induced mouse model and represents a possible therapeutic strategy for psoriasis.

## Discussion

Psoriasis is an auto-inflammatory skin disease, which seriously threaten the human health and life. It is thought to result from impaired communication between keratinocytes and immune cells, because of genetic and environmental factors [2, 6]. However, an in-depth understanding of the precise mechanism in psoriasis pathogenesis is essential for the development of new therapeutics for treating this disease.

Recently, accumulating evidence has implicated the dysregulation of the Hippo-YAP pathway in multiple human diseases, including psoriasis [10, 17, 26]. For instance, MST1, the upstream kinase of the Hippo pathway, was dramatically up-regulated in psoriasis patients’ skin lesions and T cells [18]. In addition, TEAD4, an important downstream transcription factor of the Hippo pathway, was significantly up-regulated in the psoriatic lesions compared to the healthy skins [19]. Meanwhile, Jia et al. found that YAP1, the main downstream modulator of the Hippo pathway, was up-regulated in the psoriatic skins, suggesting its possible role in psoriasis pathogenesis [26]. Our data further confirmed that YAP1 was elevated in clinical psoriatic skins (Fig. 1a, b). Moreover, a positive correlation between YAP1 expression and patient PASI scores was observed in the clinical samples (Fig. 1c). Thus, the dysregulation of YAP1 in psoriatic skins may affect the disease severity.

In psoriatic skin lesions, infiltrated immune cells produce inflammatory mediators that lead to the activation of keratinocytes [2, 6]. Interestingly, the psoriasis-related inflammatory factors, including TNF-α, IL-17, IL-22, OSM, and IL-1α, promoted YAP1 up-regulation in keratinocytes, as indicated by increased YAP1 expression (Figs. 1d, e, & f). Recently, the Shi group reported that IL-17A promoted keratinocyte proliferation through Hippo pathway [32]. Mechanistically, IL-17A stimulates the recruitment of MST1 to ACT1 in keratinocytes, leading to reduced MST1-LATS1 interaction and dephosphorylated YAP [32]. In our present study, we demonstrated that inflammatory factors not only up-regulated the transcription but also enhanced the translation of YAP1 (Fig. 1). However, the precise molecular mechanism by which psoriatic inflammation upregulates YAP1 expression is still unclear, which needs further experiments to clarify.

The *YAP1* gene is the main downstream regulator of the Hippo-YAP pathway and plays an important role in tumorigenesis [9–11]. Meanwhile, YAP1 is expressed in the skin epidermis and affects the epidermal thickness [20, 21]. However, the function of YAP1 in psoriasis has not been systematically studied. Herein, we examined its role in keratinocytes by gain or loss-of-function assay. As shown in Figs. 2 & 3, YAP1 knockdown in HaCaT cells simultaneously inhibited cellular proliferation and suppressed inflammation. On the contrary, YAP1 overexpression promoted cell proliferation and inflammatory factor production (Figs. 2 & 3). Meanwhile, pharmacological inhibition of YAP1 further confirmed that VP treatment suppressed the proliferation and the production of inflammatory factors, as well as cell cycle-related and inflammation-related signals in keratinocytes (Figs. 2 & 3). By a sideways approach, we showed that inhibition of YAP1 by VP impedes the activation of keratinocytes, demonstrating the essential role of YAP1 in psoriasis development.

We then intradermally injected si-Yap1 into mouse skin in an IMQ-induced mouse model and found that Yap1 suppression led to decreased disease severity (Figs. 4a, b, & c). Meanwhile, si-Yap1 suppressed the hyperproliferation of epidermal keratinocytes, as indicated by reduced epidermal thickening and decreased Ki67 expression (Fig. 4d, e). Additionally, si-Yap1 significantly decreased the expression of psoriasis-related genes, including *S100a7*, *S100a8*, and *S100a9* (Fig. 5a). Moreover, si-Yap1 suppressed the inflammatory genes as well as the inflammatory signals in the IMQ-induced mouse skins (Fig. 5a, b). Unexpectedly, si-Yap1 not only reduced the inflammatory response in the skin but also inhibited the systematical immune response, as demonstrated by decreased spleen and lymph node size (Fig. 5d, f), indicating its role in disrupting the positive feedback loop between epidermal keratinocytes and immune system.

The therapeutic potential of the YAP1 antagonist (VP) was then assessed on the psoriasis mouse model. As shown in Fig. 6a, b, VP treatment significantly decreased the skin inflammation and disease severity of IMQ-induced mouse skins. Additionally, VP suppressed the IMQ-induced excessive keratinocyte proliferation and inflammatory response (Fig. 6c, d, e, & f). Thus, by hindering the proliferation and inflammation, VP represents a possible therapeutic strategy for psoriasis. However, due to the poor water solubility of VP, its clinical application prospects are obviously limited. Therefore, a combination of VP and micro-needle might provide a solution, as micro-needle is less invasive and painless, whereas more direct and convenient drug delivery system [34, 35].

**Fig. 7:**
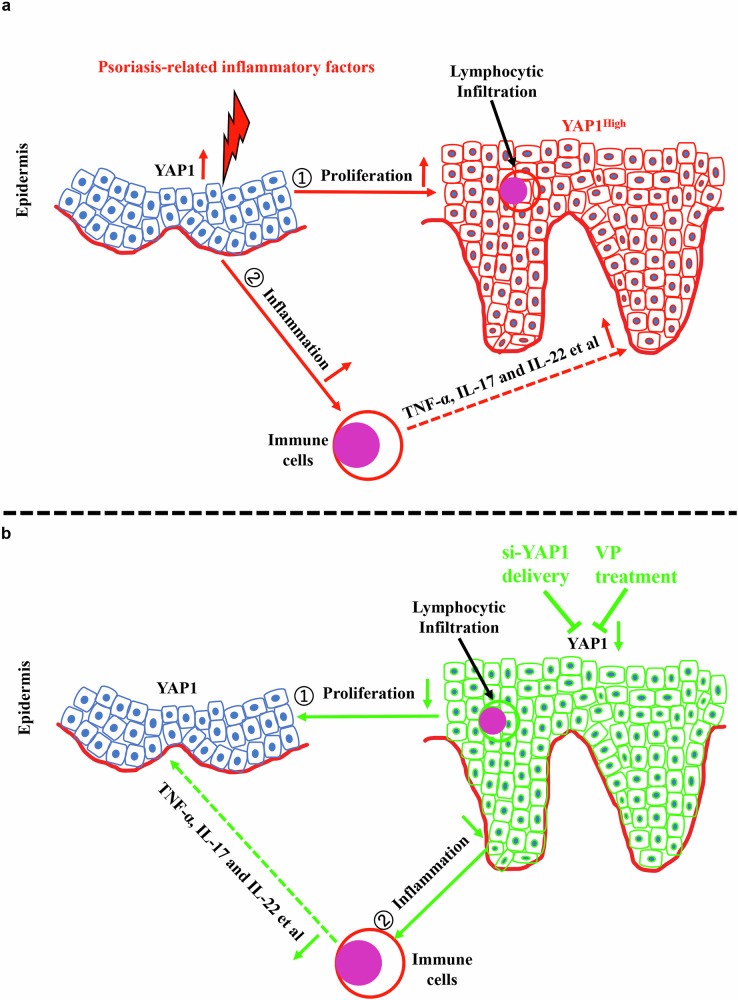
Schematic diagram of YAP1 in psoriasis pathogenesis and possible therapeutics targeting YAP1. **a** Upon being subjected to stimuli, such as psoriasis-related inflammation, YAP1 is up-regulated in keratinocytes. Up-regulated YAP1 promotes the proliferation and inflammation of keratinocytes, leading to the recruitment and activation of relevant immune cells. The activated immune cells secrete pro-inflammatory mediators (TNF-α, IL-17, and IL-22, etc.), which further cause the up-regulation of YAP1 in keratinocytes, forming a positive feedback loop and resulting in the expansion of psoriasis plaques. **b** Inhibition of YAP1 using VP/si-YAP1 leads to reduced hyperproliferation and inflammation in keratinocytes, which can disrupt the psoriasis inflammatory feedback loop, ultimately helping to enhance the therapeutic effect of psoriasis.

In conclusion, our results demonstrate that YAP1 acts as a positive regulator of psoriasis pathogenesis by promoting the proliferation and inflammation of keratinocytes. Meanwhile, stimuli, such as psoriasis-related inflammatory factors, dramatically increase YAP1 expression. Importantly, functional or pharmacological inhibition of YAP1 prevents psoriatic development in vitro and in vivo (Fig. 7), suggesting the potential of therapeutic approaches targeting YAP1 in psoriasis treatment. However, further pre-clinical or clinical studies are required to confirm the therapeutic value of targeting YAP1 in psoriasis patients.

## Supplementary information

Supplemental figure and figure legend

Supplemental Material-Original Data WB

## Data Availability

The data underlying this article will be shared on reasonable request to the corresponding author.
