# Supplementary material for: YAP1 facilitates the pathogenesis of psoriasis via modulating keratinocyte proliferation and inflammation
Source: Cell Death Dis. 2025 Mar 19;16(1):186. doi: 10.1038/s41419-025-07521-3 (PMC11923178; doi:10.1038/s41419-025-07521-3)
Supplement: Supplementary file 1 — Supplemental figure and figure legend [file 41419_2025_7521_MOESM1_ESM.pdf]

Supplemental Figure legends

Figure S1 The expression of *YAP1* in healthy controls, psoriatic lesions, and non-lesional skins was analyzed using GEO database. Analysis of *YAP1* mRNA expression in 64 normal healthy skins (NN), 58 psoriatic lesions (Involved skin lesions from psoriatic patients, PP), and 58 samples from individual-matched uninvolved skin of psoriatic patients (Uninvolved skin from psoriatic patients, PN) using the GSE13355 dataset. **: P < 0.01, compared with the indicated controls.

Figure S2. The expression of Yap1 in skin tissues derived from mouse back administrated with si-Yap1/si-CTL in the presence or absence of IMQ treatment. (a) Representative immunohistochemistry images showing the expression of Yap1 in the skin tissues derived from different groups. Scar bar: 25 μm. **(b)** Quantification data of Yap1 positive cells in the mouse skin samples of different groups (n=8 for each group). ***: P < 0.001, compared with the indicated controls.

Figure S3. The expression of Stat3 and NF-κB in the skins of mice administrated with si-Yap1 in the presence or absence of IMQ treatment. (a) Left panel: Representative images showing the Stat3 expression in the skin tissues derived from different groups. Scar bar: 25 μm. Right panel: Quantification of Stat3 positive cells in the mouse skins of different groups (n=8). **(b)** Left panel: Representative images showing the NF-κB expression in the skin tissues from different groups. Scar bar: 25 μm. Right panel: Quantification of NF-κB positive cells in the mouse skins of different groups (n=8). ***: P < 0.001, compared with the indicated controls.

Figure S4. Effects of VP on regulating systemic immune response in IMQ-induced psoriasis mouse model. (a) Representative pictures showing the spleen size of mice from different groups. **(b)** mRNA levels of *Il-1β* in the spleens derived from mice treated with VP or CTL in the presence or absence of IMQ treatment (n=8). **(c)** mRNA levels of *Il-1β*, et al. in the lymph nodes derived from different groups (n=8 for each group). ***: P < 0.001, **: P < 0.01, *: P < 0.05, compared with the indicated controls.

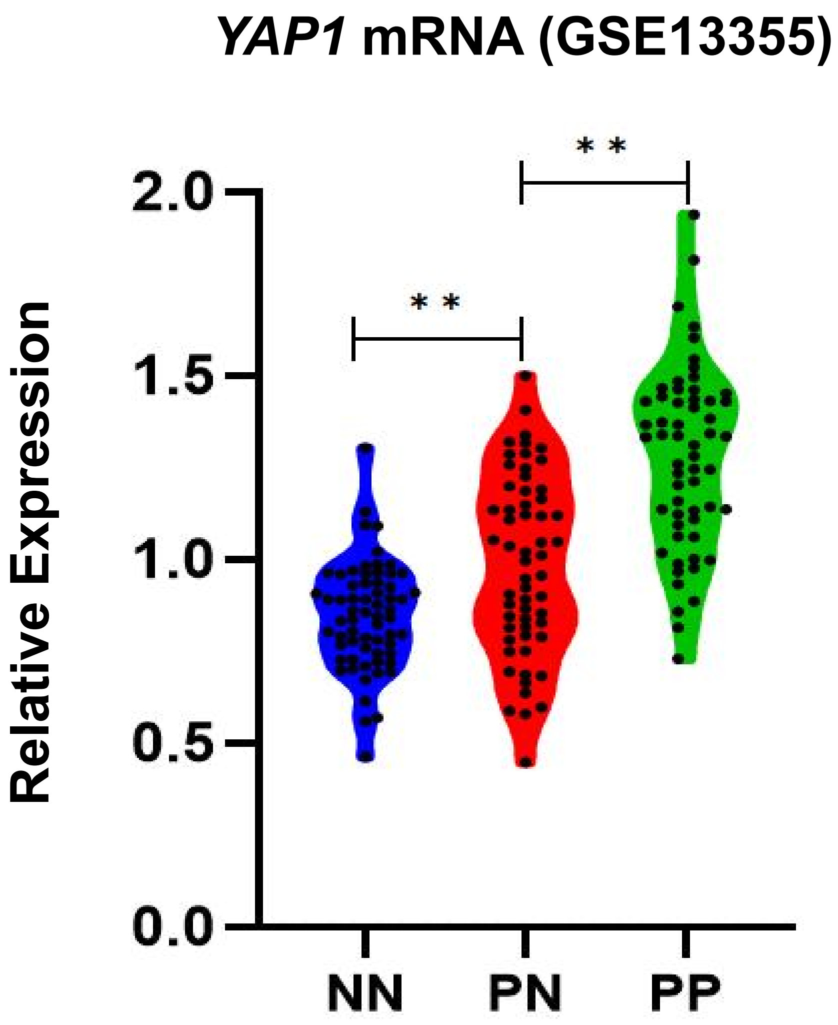

YAP1 mRNA (GSE13355)
Relative Expression
2.0
1.5
1.0
0.5
0.0
NN
PN
PP
* *
* *

Fig. S1

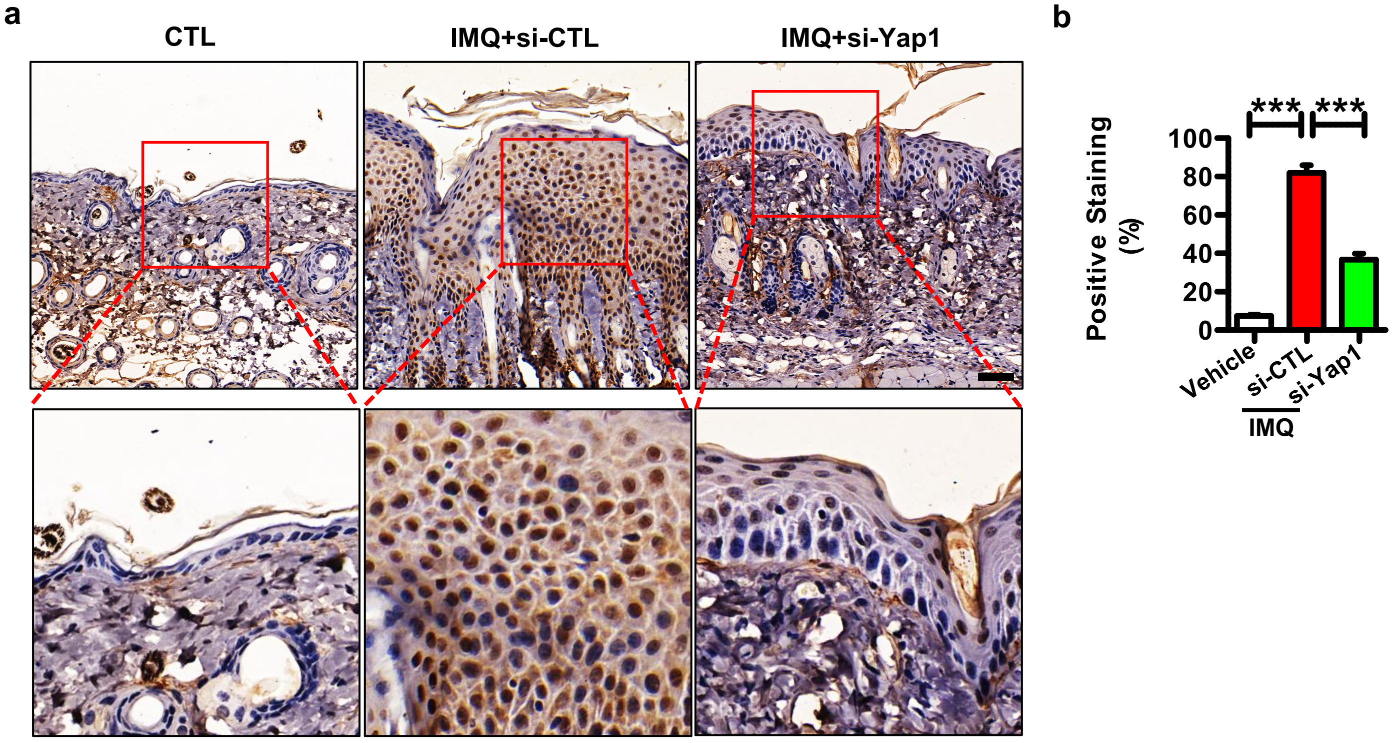
a
CTL
IMQ+si-CTL
IMQ+si-Yap1
b
Positive Staining (%)
100
80
60
40
20
0
***
***
Vehicle
si-CTL
si-Yap1
IMQ

Fig. S2

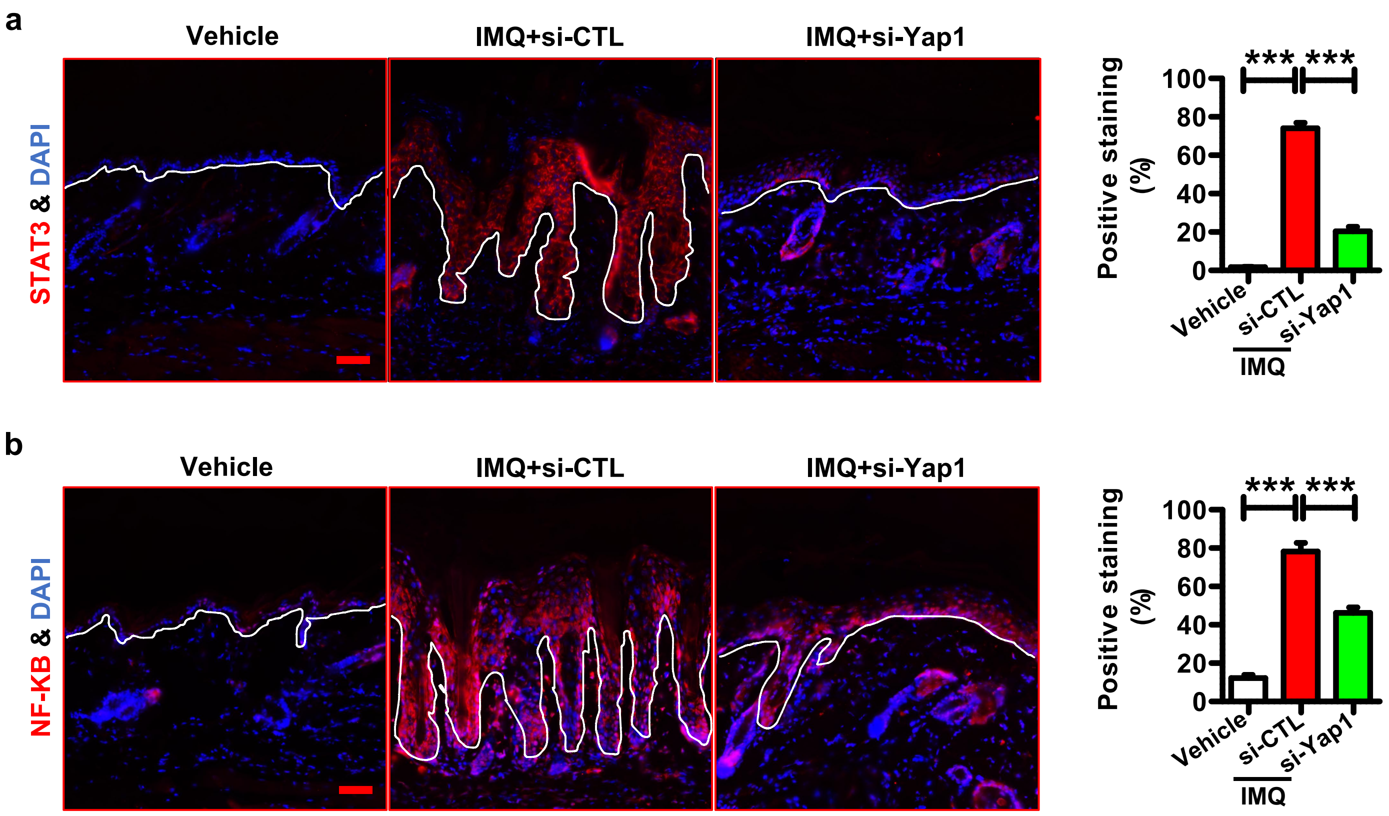
a
Vehicle
IMQ+si-CTL
IMQ+si-Yap1
STAT3 & DAPI
Positive staining (%)
***
***
100
80
60
40
20
0
Vehicle
si-CTL
si-Yap1
IMQ
b
Vehicle
IMQ+si-CTL
IMQ+si-Yap1
NF-KB & DAPI
Positive staining (%)
***
***
100
80
60
40
20
0
Vehicle
si-CTL
si-Yap1
IMQ

Fig. S3

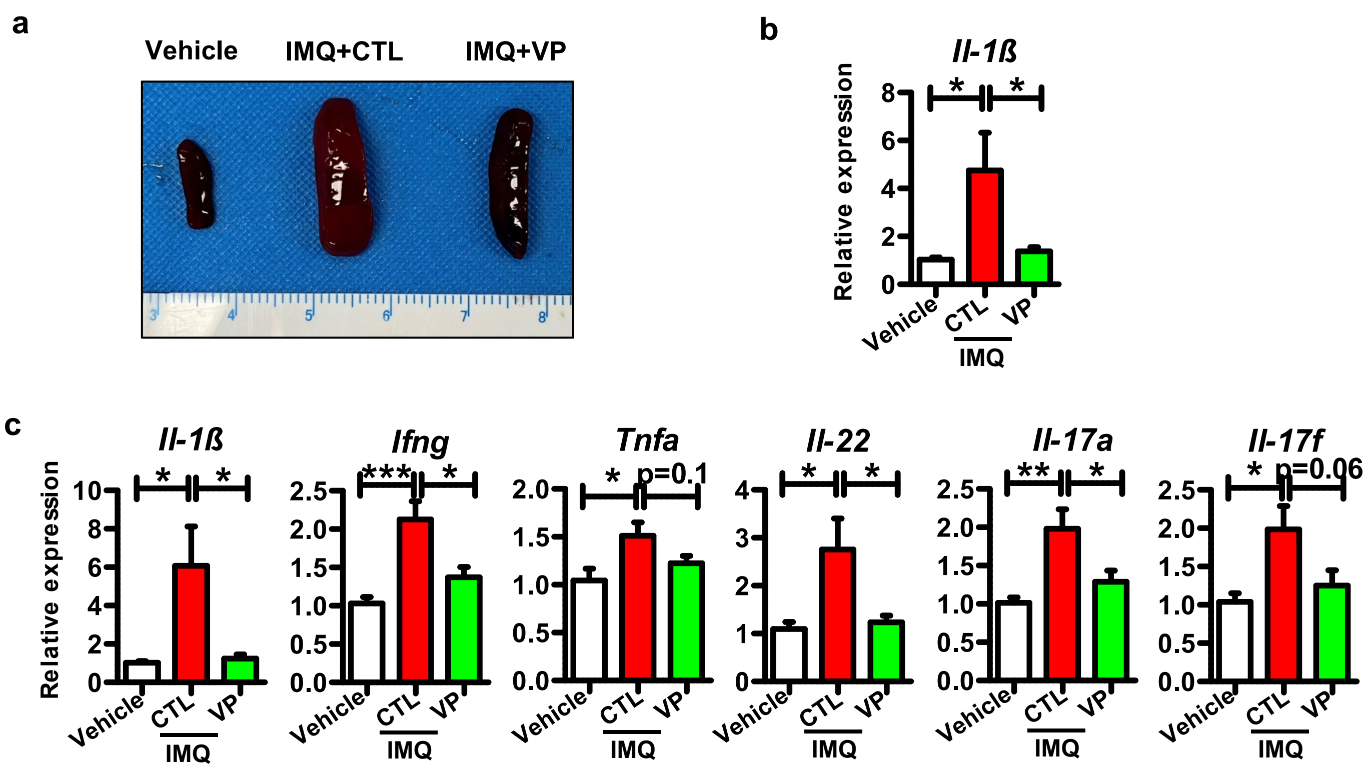
a
Vehicle
IMQ+CTL
IMQ+VP
b
Il-1ß
Relative expression
*
*
Vehicle
CTL
VP
IMQ
c
Il-1ß
Ifng
Tnfa
Il-22
Il-17a
Il-17f
***
**
p=0.1
p=0.06

Fig. S4
