# Supplementary figures and images for: YAP1 facilitates the pathogenesis of psoriasis via modulating keratinocyte proliferation and inflammation
Source: Cell Death Dis. 2025 Mar 19;16(1):186. doi: 10.1038/s41419-025-07521-3 (PMC11923178; doi:10.1038/s41419-025-07521-3)

Figure 1e

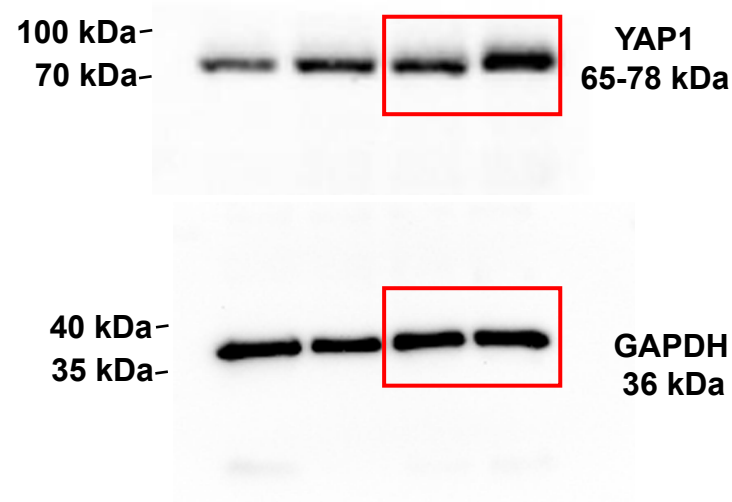

Figure 1i

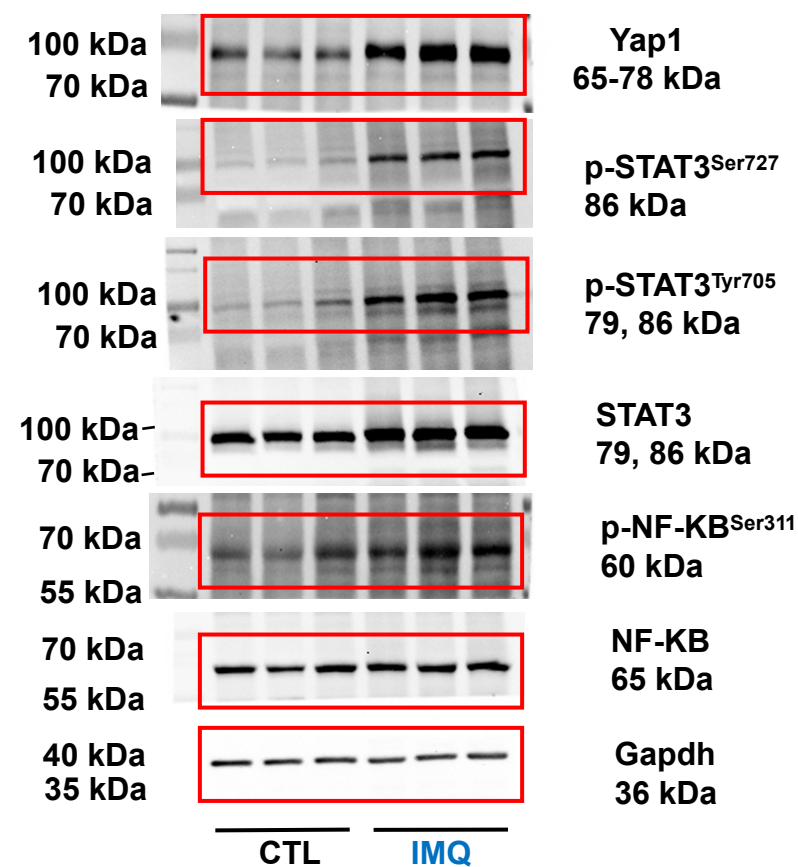

Figure 2a

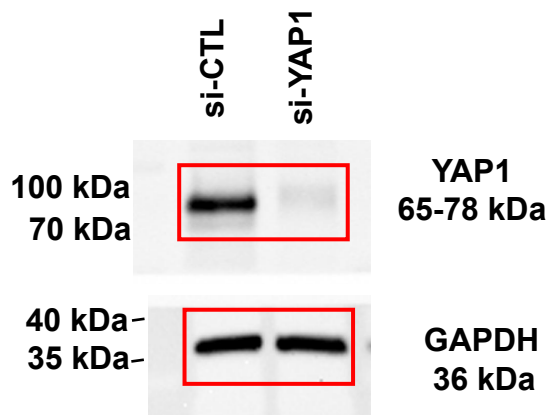

Figure 2g

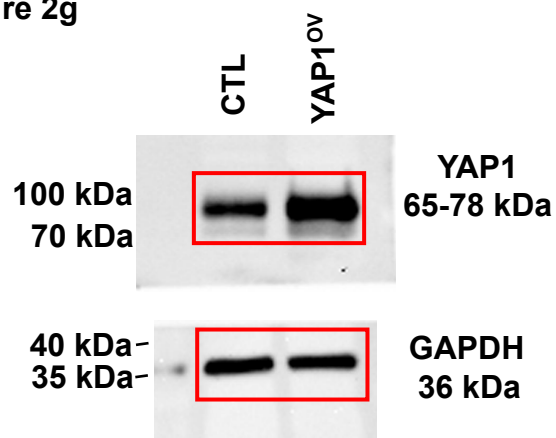

Figure 2c

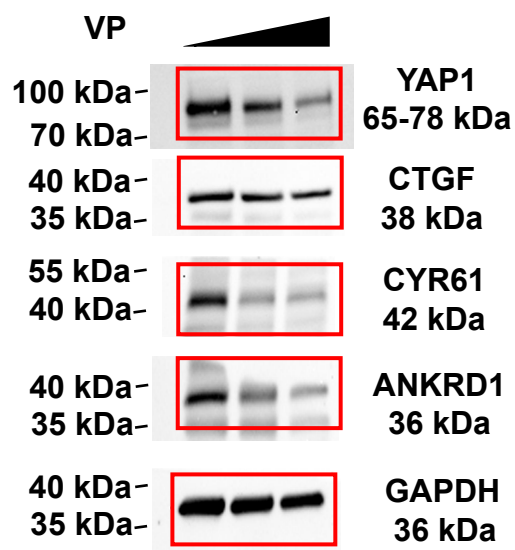

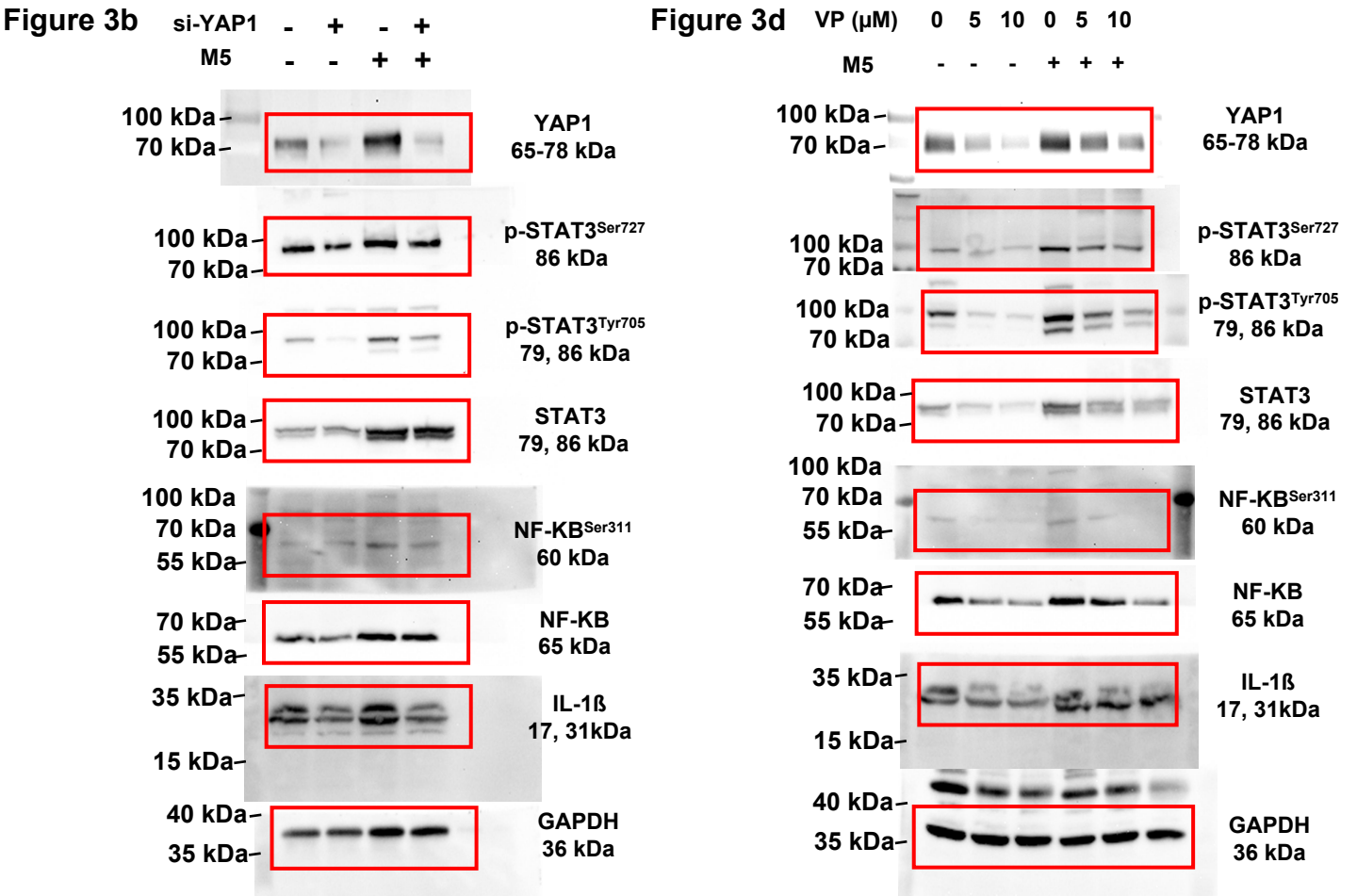

**Figure 3f**

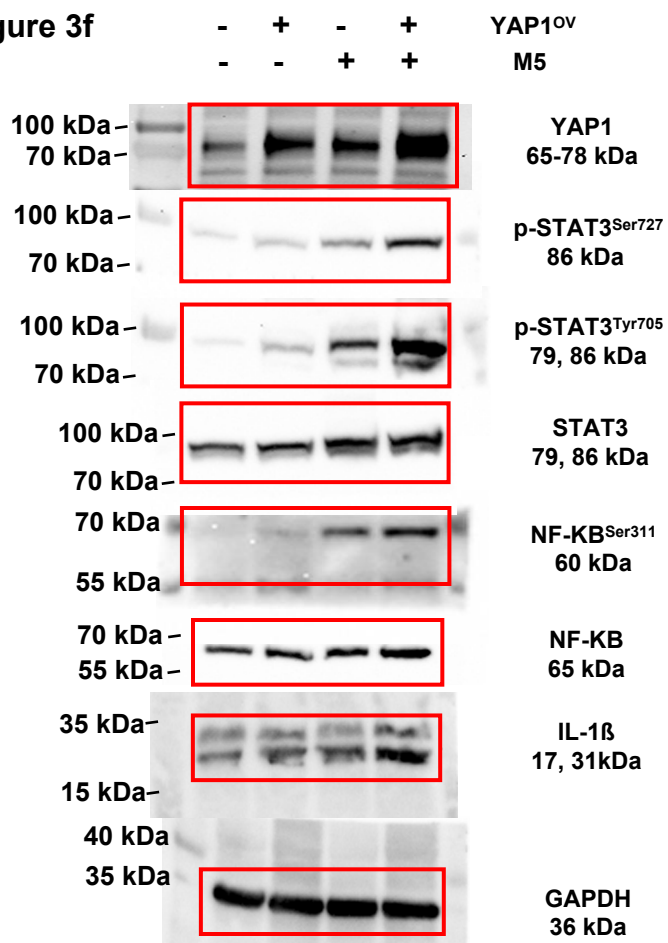

**Figure 4a**

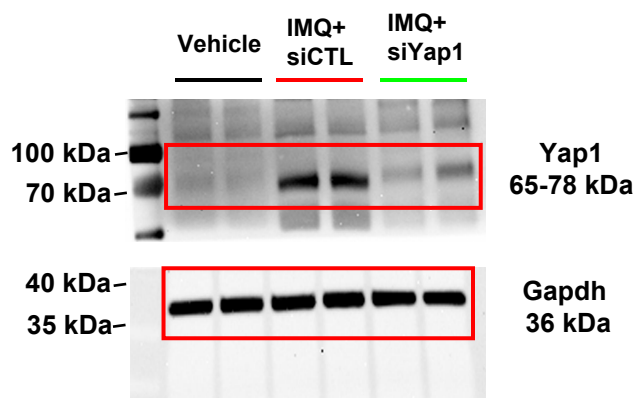

**Figure 5b**

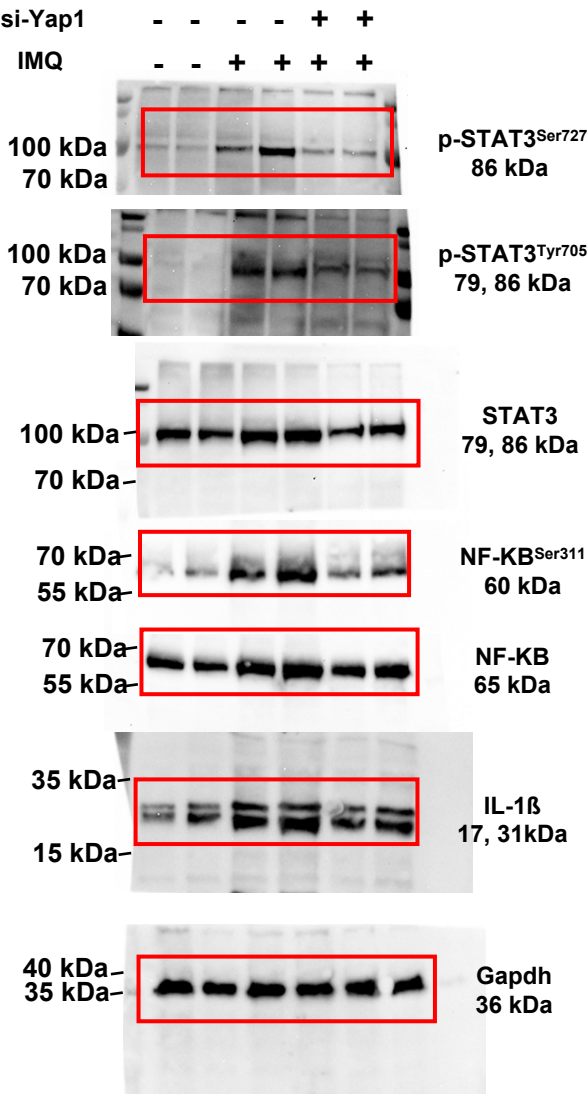

Figure 6a

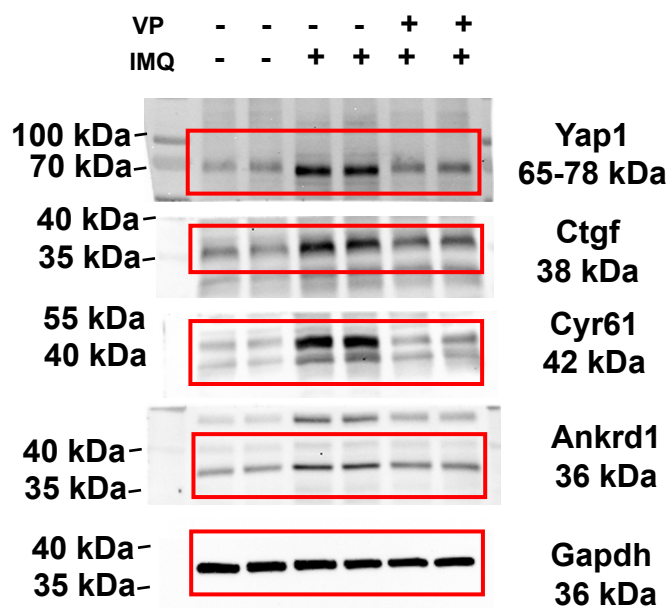

Figure 6f

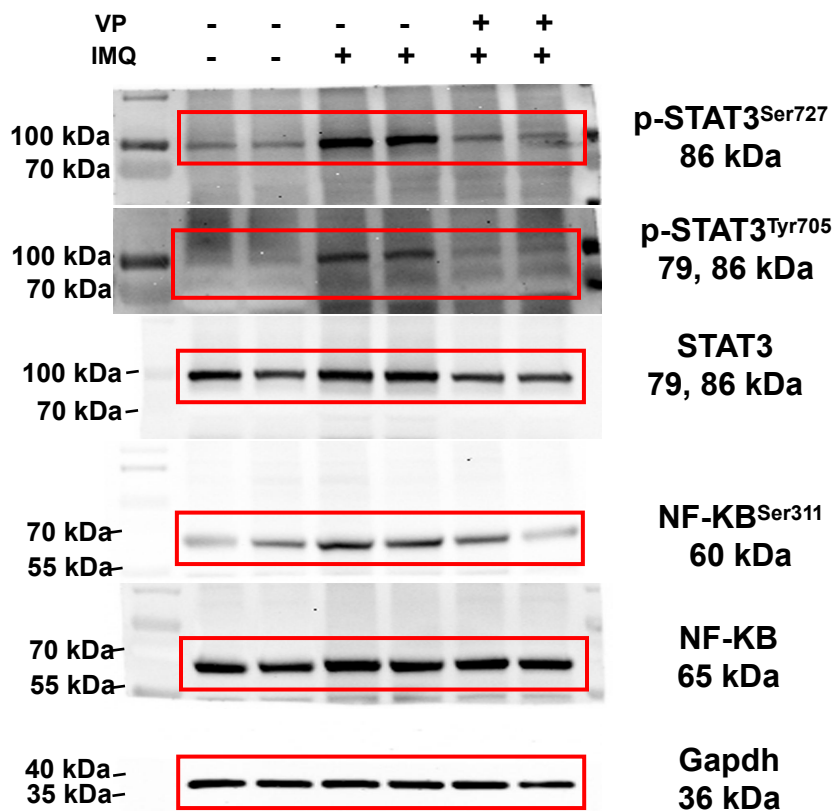

Supplement: Supplementary file 2 — Supplemental Material-Original Data WB [file 41419_2025_7521_MOESM2_ESM.pdf]
